# Coding mechanisms for main condition in ICD-11

**DOI:** 10.1186/s12911-025-03069-6

**Published:** 2025-07-10

**Authors:** Hude Quan, Olafr Steinum, Danielle A. Southern, William A. Ghali

**Affiliations:** 1 Department of Community Health Sciences, Cumming School of Medicine, University of Calgary, Calgary, Canada; 2 Nordic WHO FIC Collaborating Centre, Oslo, Norway; 3 Office of Vice President of Research & O’Brien Institute of Public Health, University of Calgary, Calgary, Canada

**Keywords:** Standards, Main condition, International classification of diseases

## Abstract

Countries have been routinely abstracting health data from hospital charts and coding conditions using ICD-10. A main condition must be assigned to each admission. However, the definition of main condition is inconsistent across countries, and may be based on (1) the initial reason for admission; (2) the reason for admission, as understood at the end of the hospital stay; and (3) the condition that consumed the most hospital resources or hospital days. Now, ICD-11 standardizes the coding schema for main condition. This paper describes the ICD-11 coding guidelines for main condition and discusses their implications for data comparability.

## Background

A patient is admitted due to upper abdominal pain. After investigation, it is determined the patient has myocardial infarction, type 1 diabetes mellitus, and essential hypertension. After three days in hospital, the patient has a stroke and remains in hospital for another month. Then the patient is discharged home.

Health care practitioners document patients’ health conditions during hospitalization, and health information management specialists (i.e., coders) read through the documents to assign International Classification of Disease (ICD) codes to those documented conditions. Among the conditions present, a main condition contributing to admission must be identified. In ICD-10, the World Health Organization (WHO) said the main condition was “defined as the condition, diagnosed at the end of the episode of health care, primarily responsible for the patient’s need for treatment or investigation” [1]. The example above highlights the distinct conceptualizations of main condition that could be applied: (1) the initial reason for admission, (2) the reason for admission, as understood at the end of the hospital stay, and (3) the condition that consumed the most hospital resources or hospital days. Each of these concepts is pertinent, and there is value to considering the diagnoses that would be selected in relation to each concept. However, hospital discharge data across countries generally identify a single diagnosis as the main condition.

Unfortunately, inconsistent definitions have been applied to the main condition in ICD-coded health data across countries [2]. For the example above, some countries, such as the United States, would record myocardial infarction as the main condition (reason for admission as understood at the end of the hospital stay), whereas other countries, such as Canada [3], would select stroke as the main condition, given it used the most resources during the hospital stay. Some countries, for instance France, have even made changes to the criteria for main condition. If disease presentation was seen as most important, the reason for admission in this example would be abdominal pain.

The variance in definitions of main condition presents challenges in determining study populations for outcomes research, healthcare system evaluation, epidemiology, and disease grouping, even if a condition could be considered both the reason for admission and the main use of resources. For example, in the study of hypertension outcomes in Canada [4], cardiovascular outcomes of myocardial infarction, heart failure, and stroke are defined based on the main condition coding field. Due to the Canadian “resource use” definition of main condition, researchers will miss patients who were admitted due to heart failure but where heart failure was not the biggest contributor to the length of stay. The number of such cases may be small, but they still have an impact.

## Main text

### Reconciling definitions of main condition in ICD-11

To address the variation, the WHO has redefined main condition as follows: “The definition of main condition relates to describing an episode of hospital-based care. Record/identify as the main condition the one condition that is determined to be the reason for admission, established at the end of the episode of health care” [1].

The WHO has revised the coding scheme by adding a new function of combining more than one code in a cluster to describe one diagnostic statement (one condition) pre-and/or post-coordination of the condition. Extension codes in Chapter X are designed to capture more clinical details through 12 head categories, such as severity scale value, aetiology, and histopathology [5]. Under each category, there are various number of subheadings. The linkage of stem code and extension codes forms a code syntax or cluster, which provides the opportunity to describe and fully characterize a documented clinical concept in detail. As a result, clinical information in administrative health data will be enriched.

### Selection of main condition

Under Diagnosis code descriptors, there are seven subheadings. One of them is discharge code descriptor. Discharge diagnosis types are specifically added as new features to specify the main condition:

- XY0Y Main condition, Reason for encounter or admission determined after study at the end of the episode.
- XY7B Main resource condition.
- XY6E Initial reason for encounter or admission.

For the opening example, the coding would look like this:

- XY0Y Main condition: myocardial infarction (BA41 Acute myocardial infarction).
- XY7B Main resource condition: stroke (8B20 Stroke not known if ischaemic or haemorrhagic).
- XY6E Initial reason for admission: upper abdominal pain (MD81.10 Pain localised to upper abdomen).
- Clustering code: BA41&XY0Y (main condition), 8B20&XY7B (main resource condition), MD81.40&XY6E (initial reason for admission).
- Other conditions: Type 1 diabetes mellitus (5A10), essential hypertension (BA00).

There are basic coding rules for using extension codes:

1. Extension codes should never be used alone and must always be linked to a stem code, which are found in 26 chapters (a tabular list) in ICD-11;
2. Stem codes are always coded before extension codes in the clustered combination of codes;
3. The ampersand (&) is used in the combining syntax to distinguish the stem code from extension codes, such as “stem code & extension code & extension code”;
4. Any correlated stem codes are linked using a forward slash (/) between stem codes, and each stem code in such complex clusters can have its own associated extension code(s) juxtaposed with a linking ampersand (&).

The new extension code scheme has advantages in data collection and analysis. ICD-11 increases the comparability of data across countries and jurisdictions. Data users can select patients for specific purposes through stem code descriptors. For example, Canada has been using resource use to determine the main condition, which is specified by diagnosis type “M” following a stem code. There is no descriptor for reason for admission.

The difficulty of selecting the main condition among those listed or described in a chart depends on the legibility and clarity of the documentation. When clinicians clearly state that a certain condition is the main factor in an episode of hospital care, coding will usually be straightforward. Sometimes, selecting the main condition is not straightforward, due to ambiguity in chart documentation. For example, a clinician may not record diagnostic information about main conditions or may record an obviously inconsistent or incorrect main condition. In this case, the record should be returned to the clinician for clarification. Where clarification is not possible, the ICD-11 reference guide presents a set of rules for the coder:

If clarification of potential erroneous documentation is not possible, one of the following rules can be applied by the clinical coder and the main condition reselected for reporting purposes. The rules are for use when the coder may be unclear as to which recorded condition should be selected as the main condition for reporting purposes.

MB1 Several conditions recorded as ‘main condition’; or

MB2 Presenting symptom of diagnosed condition recorded as ‘main condition’; or

MB3 Signs and symptoms recorded as ‘main condition’ with alternative conditions recorded as the cause.

On the ICD 11 website (https://icd.who.int/browse11/l-m/en), there are tabs for Browser, Coding Tool, Special Views and Info. The relevant extension codes for main condition and related concepts are generally easy to find in the ICD-11 Browser [6]. These are located in the chapter on extension codes (Chapter X) and can be found by browsing to the “Diagnosis code descriptors” section in the “Discharge diagnosis types” grouping. Another option is to identify relevant codes when using “main condition” or “resource” terms are searched in the Browser search field.

In the Coding Tool [7], searching key words only gives results that have specific postcoordination built-in. However, the Coding Tool provides word suggestions and completion and has a chapter filtering feature. The “Extension Codes” is filtered out by default. Importantly, however, the filter on the Coding Tool needs to be set to include the extension codes chapter. Without this setting, any search will fail to capture relevant extension code concepts.

## Discussion

ICD-coded health data have been routinely collected around the world and used widely for various purposes. But comparison of study results has been hindered by variations in how countries define and record main condition. Consider this clinical scenario: a patient admitted due to hypertension has a stroke while in hospital. Based on a “reason for admission” definition the main condition would be hypertension, but according to a “resource use” definition it would be stroke. In the latter case, rates of hypertension would be underestimated. This would have implications for the evaluation of primary and preventive care. Hypertension is one indicator of ambulatory care sensitive conditions (ACSC), and hospitalization rates for ACSC, which are calculated based on main condition, have been used in many jurisdictions as a proxy for the presence or absence of appropriate primary and preventive care [8].

Uniformity in understandings of the concept of main condition is of great significance internationally. In ICD-11, the WHO has defined the main condition as the reason for admission as determined at time of discharge. It has also created functions for capturing other main condition concepts, including main resource condition and initial reason for admission (if it differs from the final main condition), which some countries may wish to deploy as additional detail. The opportunity to capture this data is unique to ICD-11. Clustering and the new extension codes are key innovations that unlock this new potential. Importantly, the classification itself tags diagnoses (so this is not dependent on special computer systems that have particular fields for certain diagnoses). In the ACSC clinical scenario, coders will code hypertension as reason for admission and stroke as main resource condition. This method will overcome the underestimation of hypertension hospitalization rates without skewing estimates of conditions contributing to hospital resource use or case mix.

ICD-11 has specified diagnosis timing through extension code XY6M Present on admission, XY69 Developed after admission, and XY85 Uncertain timing of onset relative to admission. For diagnosis timing in surgical procedure, the extension code is XY9U Preoperative, XY9NIntraoperative and XY7V Postoperative. These diagnosis timing codes are helpful for defining main conditions with a higher likelihood of being present on admission and those that occurred during hospitalization. In the series publication related to diagnosis timing [9], the different types of diagnosis timing were examined to describe complex patients and examples of application of the ICD-11 new feature was presented.

Currently, no country is coding main condition based on both the “reason for admission” and “resource use” definitions. That said, a country could force coders to code both the main condition and the resource use without employing extension codes. However, existing case-mix systems will be affected if a country does not adopt extension codes (i.e., reason for admission and resource use) because the main condition primarily determines the grouping of related diseases. Each country will determine an implementation plan and coding guidelines for coders, but it is expected that countries will adopt the ICD-11 coding guide to enrich administrative health data. We recognize that a change to international definitions of main condition will require corresponding adjustments in case-mix systems as these are adapted to ICD-11. Of relevance to this point, there are early discussions around a WHO initiative to develop an international case-mix grouper tailored to ICD-11. The grouper is a technology of grouping patients based on ICD-11 diagnosis codes. (Time will tell if that gains traction.) The grouper is a system that gathers claim information based on diagnoses, procedures, age, sex, discharge status and the presence of complications or comorbidities. It classifies hospital cases according to groups, expected to have similar hospital resource use. Groupers can then be used to estimate healthcare resource utilization and risk adjustment analysis [10].

ICD data quality is related to the time spent on each chart. Of course, the rich detail facilitated by ICD-11 carries the risk (and price) of additional complexity. Consideration of the main resource condition and presentation at admission may add to coder burden. It will ultimately be essential for countries to determine their national health data standards, the ultimate objective for these data, and the resources required to code data well. The most worthwhile reason to code the main condition with extension codes is to leverage the data, with confidence, for international comparisons and multiple purposes.

## Data Availability

Not applicable. All examples provided in the text are from the ICD-11 Reference Guide [1].

## Declarations

### About this supplement

This article has been published as part of BMC Medical Informatics and Decision Making Volume 21 Supplement 6 2021: Spotlight on ICD-11: New Features and New Opportunities. The full contents of the supplement are available at https://bmcmedinformdecismak.biomedcentral.com/articles/supplements/volume-21-supplement-6.

## Abbreviations

AMI: Acute myocardial infarction
ACSC: Ambulatory care sensitive conditions
ICD: International Classification of Diseases
ICD-10: International Classification of Diseases, 10th revision
ICD-11: International Classification of Diseases, 11th revision
WHO: World Health Organization
